# S3 guideline diagnostics and therapy in alopecia areata – Part 2: Therapy, psychosocial and cosmetic support

**DOI:** 10.1111/ddg.70130x

**Published:** 2026-05-04

**Authors:** Ulrike Blume‐Peytavi, Andria Constantinou, Tsenka Tomova‐Simitchieva, Adrian Tanew, Michael Sticherling, Hermann Girschick, Annika Vogt, Uwe Schwichtenberg, Uwe Gieler, André Märtens, Kerstin Zienert, Claudia Stenders, Ricardo Niklas Werner, Doris Wilborn

**Affiliations:** ^1^ Department of Dermatology Venereology and Allergology Charité – Universitätsmedizin Berlin Corporate Member of Freie Universität Berlin and Humboldt‐Universität zu Berlin and Berlin Institute of Health Berlin Germany; ^2^ Private practice Vienna Austria; ^3^ Department of Dermatology University Hospital Erlangen Erlangen Germany; ^4^ Department of Pediatrics and Adolescent Medicine Vivantes Netzwerk für Gesundheit GmbH Klinikum im Friedrichshain Berlin Germany; ^5^ Derma Nord Dermatological Practice, Germany; ^6^ Psychodermatology Competence Centre University Hospital Giessen Vitos Giessen‐Marburg gGmbH Vitos Psychosomatik Giessen Giessen Germany; ^7^ Hairdresser Berlin Germany; ^8^ Alopecia Areata Deutschland e.V., Germany; ^9^ Department of Dermatology Venereology and Allergology Division of Evidence‐based Medicine (dEBM) Charité – Universitätsmedizin Berlin Corporate Member of Freie Universität Berlin and Humboldt‐Universität zu Berlin and Berlin Institute of Health Berlin Germany

**Keywords:** alopecia areata, circular hair loss, topical and systemic treatment, Quality of life

## Abstract

This second part of the S3 guideline on the diagnosis and treatment of alopecia areata (AA), presents key recommendations on topical and systemic therapy, quality of life and support services. The first part of the guideline, published separately, covers the definition and content of epidemiology and diagnosis as well as comorbidities, risk and prognostic factors.

The application of corticosteroids is the only topical intervention with a high level of recommendation. All other topical agents that can be offered for the treatment of AA have a low level of recommendation, whereby the recommendation levels for the individual topical agents vary in the age groups of children, adolescents and adults.

Of the active substances for systemic treatment of AA, only systemic corticosteroids and Janus kinase inhibitors (JAK) have a high level of recommendation, and they are restricted only to adults. Children and adolescents can be offered corticosteroids, methotrexate or JAK* (*with appropriate authorisation for the age group) for a severe form or higher. All other systemic agents should not be offered to children and adolescents. The expert group recommends that all patients with all forms of AA, regardless of age, should be informed about the possibilities of hair replacement and cosmetic options.

## INTRODUCTION

The present S3 guideline on alopecia areata (AA) consists of two separately published parts: Part two (this publication) is based on the long version dated 18 September 2025 and was developed in accordance with the AWMF guidelines version 2.1 (2023),^1^ from January 2023 to October 2025. It contains the recommendations and background texts of the chapters on therapy, quality of life, psychosocial support and cosmetic options and should be used in conjunction with Part 1 of the guideline on epidemiology and diagnostics. The guideline targets patients with AA in all age groups, and the recommendations have been formulated specifically for children, adolescents and adults.

The project on which this publication is based was funded by the Innovation Committee of the Federal Joint Committee under the funding code 01VSF22016 (S3 LL AA) in the period from 1 January 2023 to 30 June 2025. The development of the S3 guideline followed the procedure described in the AWMF regulations, version 2.0, 2021,^2^ and version 2.1, 2023.^1^

The methodological steps of guideline development were described in detail in the guideline report (see also the online supplement and the AWMF website https://register.awmf.org/de/leitlinien/detail/013‐104). The guideline is valid for five years (until September 17, 2030) and is to be updated after this period in the event of changes to key topics.

### Therapy

Topical and systemic therapies are available for the treatment of AA. Therefore, the treatment options for AA are described below separately for topical and systemic options. The guideline group has developed a flow chart according to age groups, which summarises the recommendations for diagnosis and therapy in graphic form.

**FIGURE 1:**
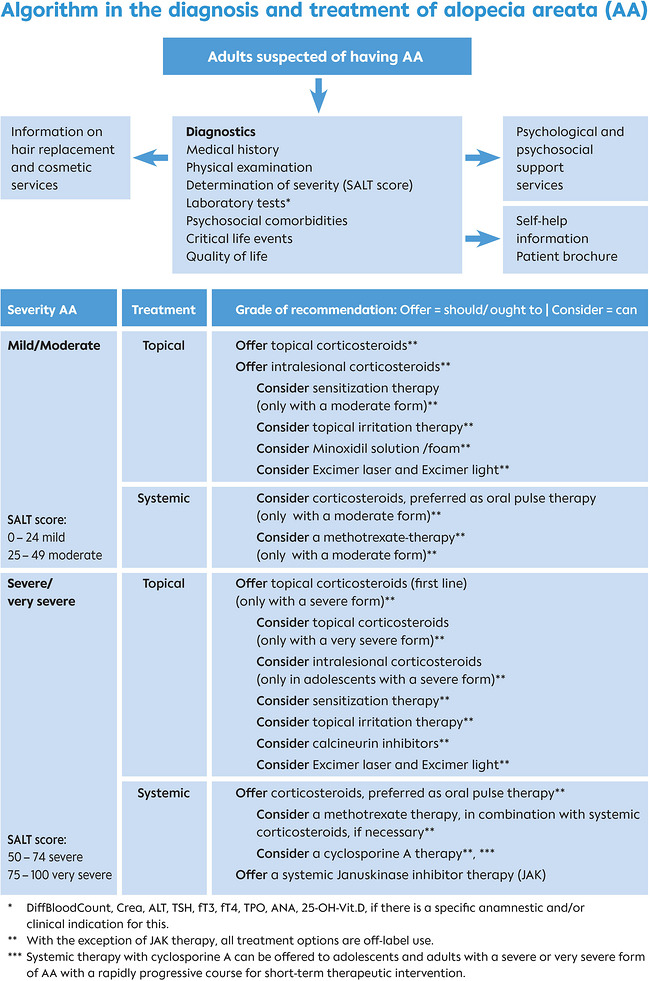
Algorithm AA for children and adolescents. [Correction added on 21 May 2026, after first online publication: Fig. 1 has been replaced.]

**FIGURE 2:**
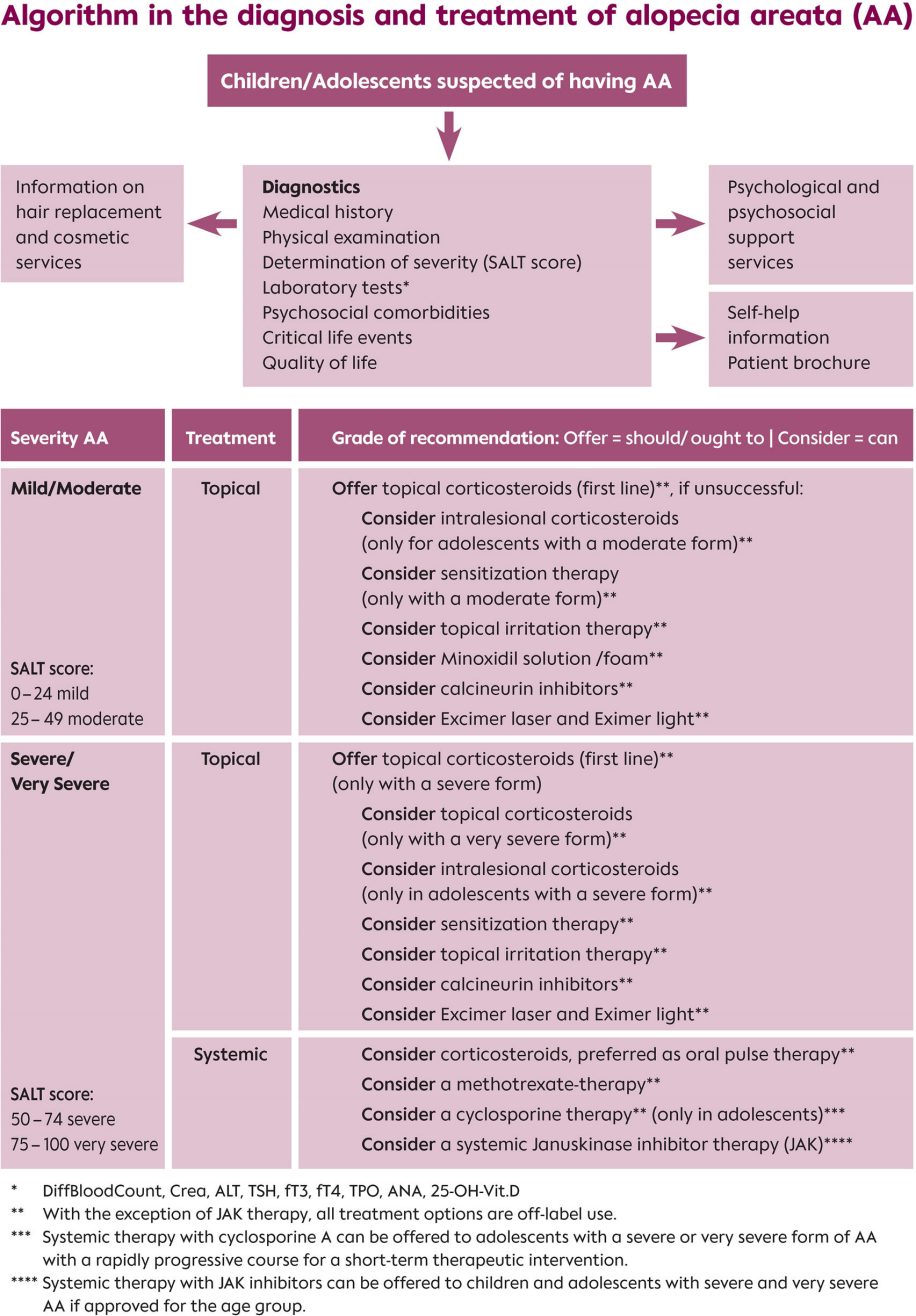
Algorithm AA for adults. [Correction added on 21 May 2026, after first online publication: Fig. 2 has been replaced.]

Figures 1 and 2 below show the treatment options for the mild/moderate and severe/very severe severity levels of AA, broken down by age group. The wording “offer” reflects the recommendation strength “should” or “ought to”. The term “consideration” indicates an “optional *can‐recommendation*”.

Table 1 shows all topical treatment options, separated for age group and severity of AA with different levels of recommendation. Severity of AA is indicated by SALT score (Severity of Alopecia Tool).

**TABLE 1:** Overview of all recommendations by active substance ‐ Topical therapy

Active substance	Severity SALT‐Score	Children	Adolescents	Adults
**Topical application of corticosteroids**	Mild <25%	↑↑	↑↑	↑↑
	Moderate 25‐49%	↑↑	↑↑	↑↑
	Severe >50‐75%	↑	↑↑	↑↑
	Very severe 76% ‐ 100%	0	0	0
	Eyebrows, beard	↑	↑	↑
**Intralesional application of corticosteroids**	Mild <25%	↓	↓	↑
	Moderate 25‐49%	↓	0	↑
	Severe >50‐75%	↓	0	0
	Very severe 76% ‐ 100%	↓	↓	↓
	Eyebrows, beard	No recommendation	0^*^	0^*^
**Contact‐Immunotherapy (DCP/SADBE)**	Mild <25%	↓↓	↓↓	↓↓
	Moderate 25‐49%	0	0	0
	Severe >50‐75%	0	0	0
	Very severe 76% ‐ 100%	0	0	0
**Stimulation therapy with Dithranol**	Mild <25%	0	0	0
	Moderate 25‐49%	0	0	0
	Severe >50‐75%	0	0	0
	Very severe 76% ‐ 100%	0	0	0
**Minoxidil Solution or foam**	Mild <25%	0	0	0
	Moderate 25‐49%	0	0	0
	Severe >50‐75%	No recommendation	No recommendation	No recommendation
	Very severe 76% ‐ 100%	No recommendation	No recommendation	No recommendation
**Calcineurin‐Inhibitors**	Mild <25%	0	0	No recommendation
	Moderate 25‐49%	0	0	No recommendation
	Severe >50‐75%	0	0	0
	Very severe 76% ‐ 100%	0	0	0
	Eyebrows, beard	↑	↑	↑
**Prostaglandin F2α‐Analogues**	Mild <25%	↓↓	↓↓	No recommendation
	Moderate 25‐49%	↓↓	↓↓	No recommendation
	Severe >50‐75%	↓↓	↓↓	No recommendation
	Very severe 76% ‐ 100%	↓↓	↓↓	No recommendation
	Eyelashes	0	0	0
**Platelet‐rich plasma (PRP)**	Mild <25%	↓↓	↓↓	No recommendation
	Moderate 25‐49%	↓↓	↓↓	No recommendation
	Severe >50‐75%	↓↓	↓↓	No recommendation
	Very severe 76% ‐ 100%	↓↓	↓↓	No recommendation
**JAK‐Inhibitors**	Mild <25%	↓↓	↓↓	↓↓
	Moderate 25‐49%	↓↓	↓↓	↓↓
	Severe >50‐75%	↓↓	↓↓	↓↓
	Very severe 76% ‐ 100%	↓↓	↓↓	↓↓
**Excimer Laser / Excimer Light**	Mild <25%	0	0	0
	Moderate 25‐49%	0	0	0
	Severe >50‐75%	0	0	0
	Very severe 76% ‐ 100%	0	0	0
**UVA1 Phototherapy and Photochemotherapy (PUVA)**	Mild <25%	↓	↓	↓
	Moderate 25‐49%	↓	↓	↓
	Severe >50‐75%	↓	↓	↓
	Very severe 76% ‐ 100%	↓	↓	↓

* Only offered by doctors experienced in the application

### Watch‐and‐wait

The watch‐and‐wait approach is described in detail in Chapter 5.1 of the long version of the guideline. The guideline group has not formulated any recommendations for this approach.

### Topical therapy

#### Introduction

Various active substances used in topical application forms in the treatment of AA are generally not authorised for the treatment of AA in Germany but can be used in *off‐label‐use*. Reference is made to *off‐label‐use* in each recommendation. However, it is not mentioned again in the background text of the long version. According to the Social Safety Code (SGB) Fifth Book (V) ‐ Statutory Health Insurance ‐ (Article 1 of the Act of 20 December 1988, Federal Law Gazette I p. 2477), § 34 Excluded drugs, remedies and aids, all drugs that “serve to improve hair growth” are affected by this regulation, which means that the statutory health insurance funds do not cover the costs of these drugs in the treatment of AA.^3^ To have the costs covered for children, adolescents and adults, individual applications for reimbursement can be submitted to health insurance companies. However, due to the legal situation described above, most individual applications for reimbursement are rejected by health insurance companies.

Recommendations for all active ingredients of topical therapy are presented here, but only selected therapies are described in detail as in the long version of the guideline. In the detailed description, we focus on newer therapies such as platelet‐rich plasma (PRP), light and laser therapy and topical Janus kinase inhibitors.

### General principles of therapy with corticosteroids

**Table:** 

	General principles of therapy with corticosteroids	Recommendation strength
**E1**	In case of nonresponse, treatment with topical or intralesional corticosteroids **ought to** not exceed a treatment period of 3 months.	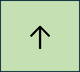
**E2**	If there is a therapeutic response to topical or intralesional corticosteroids, this therapy **ought to** be continued on an individualised basis with a maximum duration of 12 months.	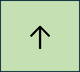
**E3**	For topical treatment of AA of the scalp with corticosteroids, substances of active substance classes III‐IV (according to Niedner) with a high therapeutic index **should** preferably be used in adolescents and adults.	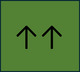
**E4**	For topical treatment of AA of the scalp with corticosteroids, substances of drug classes II‐III (according to Niedner) with a high therapeutic index **should** preferably be used in children.	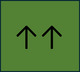
**E5**	For intralesional therapy of AA of the scalp with corticosteroids, 2.5‐10 mg/mL triamcinolone acetonide (0.1 mL / cm^2^, max. 100 cm^2^, strictly intracutaneous) **should** be used.	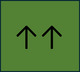
	Strong consensus	

Note: recommendations for topical corticosteroids refer to application to the scalp. Recommendations for specific localisations are described separately.

### Topical corticosteroids

**Table:** 

	Recommendation	Recommendation strength
**E34**	Topical application of corticosteroids **should be** offered to children with a mild or moderate form of AA and to adolescents and adults with a mild, moderate or severe form of AA in *off‐label‐use*.	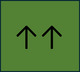
	Strong consensus	**Evidence‐based**
	References: ^4^, ^5^, ^6^, ^7^, ^8^, ^9^, ^10^, ^11^, ^12^, ^13^, ^14^, ^15^	
	LoE: Level 2*, Level 2	
	Level 2*: systematic review downgraded from level 1 to level 2, or meta‐analysis if only observational studies were included (see Oxford 2011 LoE scheme)^16^	

**Table:** 

	Recommendation	Recommendation strength
**E35**	Topical application of corticosteroids **ought to** be offered to children with a severe form of AA in *off‐label‐use*.	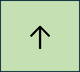
	Strong consensus	**Evidence‐based**
	References: ^4^	
	LoE: Level 2*	

**Table:** 

	Recommendation	Recommendation strength
**E36**	Topical application of corticosteroids **can** be offered to children with a very severe form of AA with nail involvement and/or body hair loss in *off‐label‐use*.	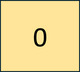
	Consensus	**Evidence‐based**
	References: ^4^	
	LoE: Level 2*	

**Table:** 

	Recommendation	Recommendation strength
**E37**	Topical application of corticosteroids **can** be offered to adolescents and adults with a very severe form of AA with nail involvement and/or body hair loss in *off‐label‐use*.	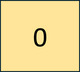
	Strong consensus	**Consensus‐based**
	LoE: Level 2*	

The full explanations on efficacy, dosage and special instructions can be found in the long version of the guideline.

### Intralesional corticosteroids

Note: recommendations for intralesional corticosteroids (ILC) refer to application to the scalp. Recommendations for specific localisations are described separately.

**Table:** 

	Recommendation	Recommendation strength
**E38**	Intralesional application of corticosteroids **should not** be offered to children with all forms of AA and adolescents with a mild form of AA.	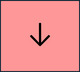
	Strong consensus	**Evidence‐based**
	References: 4, 17	
	LoE: Level 2*, Level 4	

**Table:** 

	Recommendation	Recommendation strength
**E39**	The intralesional application of corticosteroids **can** be offered to adolescents with a moderate or severe form of AA in *off‐label‐use*.	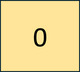
	Strong consensus	**Evidence‐based**
	References: ^18^	
	LoE: Level 2	

**Table:** 

	Recommendation	Recommendation strength
**E40**	Intralesional application of corticosteroids **should not** be offered to adolescents and adults with a very severe form of AA.	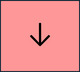
	Strong consensus	**Consensus‐based**

**Table:** 

	Recommendation	Recommendation strength
**E41**	The intralesional application of corticosteroids **ought to** be offered to adults with a mild or moderate form of AA in *off‐label‐use*.	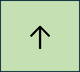
	Strong consensus	**Evidence‐based**
	References: 18, 19, 20, 21, 22, 23, 24	
	LoE: Level 2	

**Table:** 

	Recommendation	Recommendation strength
**E42**	The intralesional application of corticosteroids **can** be offered to adults with a severe form of AA in *off‐label‐use*.	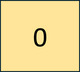
	Strong consensus	**Consensus‐based**

The full explanations on efficacy, dosage and special instructions can be found in the long version of the guideline.

### Topical immunotherapy

**Table:** 

	Recommendation	Recommendation strength
**E43**	Contact immunotherapy (DCP/SADBE) **shall not** be offered to children, adolescents and adults with a mild form of AA.	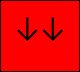
	Strong consensus	**Consensus‐based**

**Table:** 

	Recommendation	Recommendation strength
**E44**	Contact immunotherapy (DCP/SADBE) **can** be considered for children with a moderate, severe or very severe chronic form of AA as an individualised treatment trial in *off‐label‐use*.	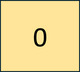
	Strong consensus	**Evidence‐based**
	References: 4, 25, 26, 27, 28, 29, 30, 31, 32	
	LoE: Level 2*, Level 2	

**Table:** 

	Recommendation	Recommendation strength
**E45**	Contact immunotherapy (DCP/SADBE) **can** be offered to adolescents and adults with a moderate, severe or very severe form of AA as an individualised treatment trial in *off‐label‐use*.	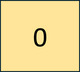
	Strong consensus	**Evidence‐based**
	References: 4, 25, 26, 27, 28, 29, 30, 31, 32	
	LoE: Level 2*, Level 2	

The manufacturer of diphenylcyclopropenone (DCP) active ingredient for use in humans has discontinued production in Germany, resulting in restricted availability currently. It is not yet known whether another supplier will resume production of this active ingredient for use in humans.

Full explanations on efficacy, dosage and special instructions can be found in the long version of the guideline.

### Topical stimulation therapy

**Table:** 

	Recommendation	Recommendation strength
**E46**	Stimulation therapy with dithranol **can** be offered to children and adolescents with all forms of AA in *off‐label‐use*.	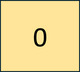
	Strong consensus	**Evidence‐based**
	References: 4, 25	
	LoE: Level 2*	

**Table:** 

	Recommendation	Recommendation strength
**E47**	Stimulation therapy with dithranol **can** be offered to adults with all forms of AA in *off‐label‐use*.	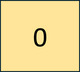
	Consensus	**Evidence‐based**
	References: 32, 33	
	LoE: Level 2	

Full explanations on efficacy, dosage and special instructions can be found in the long version of the guideline.

### Topical minoxidil

**Table:** 

	Recommendation	Recommendation strength
**E48**	The use of minoxidil solution or foam **can** be offered to children, adolescents and adults with mild or moderate AA in *off‐label‐use*.	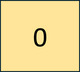
	Strong consensus	**Evidence‐based**
	References: 4, 29, 34, 35, 36	
	LoE: Level 1, Level 2*, Level 2	

Full explanations on efficacy, dosage and special instructions can be found in the long version of the guideline.

### Topical calcineurin inhibitors

**Table:** 

	Recommendation	Recommendation strength
**E49**	The topical application of calcineurin inhibitors **can** be offered to children and adolescents with all forms of AA and adults with severe or very severe AA in *off‐label‐use*.	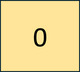
	Strong consensus	**Evidence‐based**
	References: 4, 7, 9, 10, 11, 12, 37	
	LoE: Level 2*, Level 2	

Full explanations on efficacy, dosage and special instructions can be found in the long version of the guideline.

### Topical application of prostaglandin F2α analogues

**Table:** 

	Recommendation	Recommendation strength
**E50**	Topical application of prostaglandin F2α analogues **shall not** be offered to children, adolescents and adults with all forms of AA for application to the scalp.	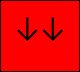
	Strong consensus	**Evidence‐based**
	References: 38, 39	
	LoE: Level 2	

Full explanations on efficacy, dosage and special instructions can be found in the long version of the guideline.

### Platelet‐rich plasma (PRP)

**Table:** 

	Statement	
**S2**	Based on the available studies, no recommendation can be made for or against the use of PRP in AA.	
	Strong consensus	**Evidence‐based**
	References: 31, 40, 41, 42	
	LoE: Level 1, Level 2	

**Table:** 

	Recommendation	Recommendation strength
**E51**	Children and adolescents **shall not** be offered PRP for the treatment of AA.	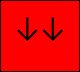
	Strong consensus	**Evidence‐based**
	References: 40, 41	
	LoE: Level 1	

The full explanations on efficacy, dosage and special instructions can be found in the long version of the guideline.

### Excerpt from mechanisms of action and efficacy

A meta‐analysis of four included RCTs with a total of 201 patients comparing intralesional PRP therapy with intralesional triamcinolone acetonide (TA) therapy shows that a mean difference (MD) in the SALT score of MD ‐2.04 (95% CI ‐4.72 ‐ 0.65) was achieved for TA after twelve weeks. The meta‐analysis shows a superiority of TA treatment compared to PRP, but the result was not statistically significant.^40^

In the systematic review by Tejapira et al. 2022 with 23 studies and 621 patients, PRP showed superiority over placebo in mild cases of AA in terms of reducing the SALT score, hair regrowth and the reduction of dystrophic hair. In more severe cases of AA (AT), however, PRP as monotherapy proved ineffective.^41^ Compared to TA, five studies show comparable efficacy of PRP in patients with AA < 25% or disease duration < 6 months. PRP was superior to topical 5% minoxidil in improving dystrophic hair and had a greater effect on hair diameter compared to low level laser therapy (LLLT).^41^

In another RCT that was not included in the meta‐analysis by Meznerics et al. 2022, PRP therapy was also found to be less effective than intralesional TA. In the TA group, 90% of patients responded to treatment, while in the PRP group 73% of patients showed a response (p<0.05).^42^ The combination of PRP with DCP showed no superiority over DCP monotherapy.^31^

Tejapira et al. 2022 critically discuss the following disadvantages in the studies included in their review: they consider the short follow‐up period of the studies to mean that the safety of PRP therapy cannot be adequately assessed.^41^ Evidence showing the long‐term efficacy of the therapy is still lacking, as well as suitable criteria for selecting patients who are suitable for the therapy. Future studies should focus on the current heterogeneity in PRP therapy.

Despite promising results in some studies, the clinical application of PRP is hampered by the lack of consensus regarding preparation and treatment protocols, the number and interval of sessions and dosing.^41^ Overall, PRP is a potential treatment option for AA, especially in mild cases.

RCTs on the efficacy of PRP in children were not identified in the literature search. Due to the multiple injections and painfulness and the lack of clear efficacy in children and adolescents, PRP should not be indicated in this age group.

### Topical JAK inhibitors

**Table:** 

	Recommendation	Recommendation strength
**E52**	Topical JAK inhibitors **shall not** be offered for the treatment of AA.	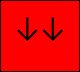
	Strong consensus	**Evidence‐based**
	References: 25, 29, 43, 44, 45, 46	
	LoE: Level 1	

The full explanations on efficacy, dosage and special instructions can be found in the long version of the guideline.

### Excerpt from Mechanisms of action and efficacy

Janus kinase inhibitors (JAK inhibitors) are a new class of drugs that interfere with cytokine signalling and thus inhibit inflammatory processes. In the case of AA, it is assumed that JAK inhibitors reduce cytokine‐mediated inflammation of the hair follicles and thus promote hair growth.^47^

The efficacy of topical JAK inhibitors in AA has been described in several meta‐analyses and in the Cochrane Review by Mateos‐Haro et al. 2023. The Cochrane Review summarises all JAK inhibitors in topical application and evaluates them in terms of hair regrowth of at least 75% after 12 to 26 weeks (RR: 5.00; 95% CI 0.25‐100.89).^29^ Compared to placebo, regrowth was five times better, but the results are not statistically significant, and the very wide confidence interval significantly limits confidence in the effect estimate.^29^

### Delgocitinib

In an exploratory double‐blind study with 31 patients with moderate to severe AA (≥30% scalp involvement), there was no significant improvement in the percentage of patients who achieved a 50% reduction in the SALT score after twelve weeks of treatment with delgocitinib ointment (30 mg/g, twice daily) compared to a vehicle ointment.^48^

### Topical ruxolitinib

In a meta‐analysis, randomised controlled trials (RCTs) showed no superiority of the topical JAK inhibitor ruxolitinib over placebo in terms of a 50% improvement in the SALT score (RR: 1.00; 95% CI: 0.31–3.18). In non‐RCTs, seven out of 22 patients on topical ruxolitinib or tofacitinib (a selective JAK1‐JAK3 inhibitor) achieved at least 50% improvement in SALT score (0.28; 95% CI: 0.01‐0.72).^43^ In a double‐blind clinical trial, 78 AA patients (SALT ≥25) were randomised to apply 1.5% ruxolitinib cream (n = 39) or vehicle cream (n = 39) twice daily to the affected scalp region and up to 2.5 cm into the surrounding areas for a total of 24 weeks. At week 24, no statistically significant difference was observed between the two groups, 12.8% (n = 5/39) of patients achieved a 50% improvement in SALT score after application of ruxolitinib cream or vehicle cream.^49^ Although pilot studies prior to clinical trials (RCT) have shown the efficacy of ruxolitinib in the treatment of AA, the results of the RCTs do not show satisfactory efficacy of ruxolitinib cream.

A subgroup analysis in a meta‐analysis found that good or complete hair regrowth was achieved in an average of 20.8% of patients on topical tofacitinib therapy compared to 46.5% on oral therapy, although this difference was not statistically significant (p = 0.19).^45^ In children with AA, a meta‐analysis found a hair regrowth event rate of 23% (95% CI: 4‐48%) in 17 children receiving topical tofacitinib 2%.^25^ In another meta‐analysis of 69 children, 71% of children responded to topical JAK inhibitor therapy, while 28% showed no response.^44^

### Special notes

The evidence on the efficacy of topical JAK inhibitors in AA is limited and partly contradictory. Most studies were observational studies or case series without control groups, which limits the validity of the results. In addition, four out of eight registered clinical trials on topical JAK inhibitors in AA were terminated prematurely due to lack of efficacy.^46^

### Laser therapies

This section describes the following laser procedures: excimer laser, excimer light, fractional CO2 laser, and low‐level laser therapy (LLLT). Studies that met the inclusion criteria were identified in the literature search for these procedures. No other laser procedures were identified in the search for the treatment of AA. Laser procedures such as excimer laser or excimer light, fractional CO2 laser, and low‐level laser therapy are used as non‐invasive treatment options.

**Table:** 

	Recommendation	Recommendation strength
**E53**	The use of targeted UVB phototherapy (excimer laser or excimer light) **can** be considered for *off‐label‐use* in children, adolescents and adults with all forms of AA.	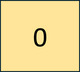
	Strong consensus	**Evidence‐based**
	References: 4, 25, 50, 51, 52, 53	
	LoE: Level 1, Level 2*, Level 2	

**Table:** 

	Statement	
**S3**	Based on the evidence identified, the Guideline Group cannot recommend the use of high‐energy and low‐level laser systems (except for excimer therapy, see recommendation E 53) for the treatment of AA in children, adolescents and adults.	
	Strong consensus	**Evidence‐based**
	References: 4, 25, 51, 52, 54, 55, 56	
	LoE: Level 1, Level 2*, Level 2	

The full explanations on efficacy, dosage and special instructions can be found in the long version of the guideline.

### Excerpt from mechanisms of action and efficacy

Here are some pointers to help distinguish between the technologies identified: Low‐level laser therapy (LLLT), also known as cold laser therapy, operates at lower power levels and relies primarily on photochemical effects to stimulate cellular repair mechanisms. Class IV laser therapy, on the other hand, uses higher power densities to achieve deeper tissue penetration and can produce both photochemical and photothermal effects.^57^

LLLT is a FDA‐approved treatment for androgenetic alopecia (AGA). It uses red light to stimulate cell metabolism, move the hair follicles into the anagen phase (growth phase) and prolong this phase, which promotes hair growth.^58^

In ablative fractional laser resurfacing, laser energy is used to create microthermal zones that cause skin remodelling without scarring through rapid wound healing. The process includes inflammation, cell migration and the formation of tissue such as neocollagen. Erbium‐doped yttrium aluminium garnet (Er:YAG) and carbon dioxide (CO_2)_ lasers are used, with programmable parameters controlling the coagulation area, penetration depth and recovery time.^58^

Excimer lasers, which generally use a combination of a noble gas (such as argon or xenon) and a halogen (such as chlorine or fluorine), generate ultraviolet (UV) light with a wavelength of 308 nm. In addition to the excimer laser, there are now also excimer light systems not based on laser technology, which also emit high‐intensity light with a maximum at 308 nm. It is assumed that excimer laser/light therapy triggers apoptosis (programmed cell death) in T cells. By reducing the number of T cells and antigen presentation, excimer irradiation may help to stabilise immune‐mediated skin diseases such as psoriasis and AA.^59^

The meta‐analysis by Lee et al. 2020,^50^ involving 129 AA patients based on nine prospective studies, four of which had no control group, examined the efficacy and safety of excimer laser or excimer light therapy compared to placebo treatment. In five studies, the treatment was administered twice a week, in two studies once a week, and in two studies every two weeks. The median treatment duration was 12 weeks, ranging from 4 to 62 weeks. 50.2% (95% CI: 31.5%‐68.9%) of patients achieved hair regrowth of more than 75% under excimer therapy. In patients with localised AA, the rate was 56.8% (95% CI: 34.7%‐79.1%). The probability of hair regrowth of more than 75% was 7.8 times higher with laser therapy than with placebo (RR: 7.83; 95% CI: 2.11‐29.11).^50^ The meta‐analysis calculations included data from excimer laser treatment and excimer light treatment without differentiating between the two methods.^50^

A randomised clinical study from 2022, which is not included in the above meta‐analysis, compares the efficacy of the 308 nm excimer laser procedure (XTRAC®; Ultra PhotoMedex) with the administration of intralesional corticosteroids.^53^ The skin areas treated with the excimer laser were irradiated at regular intervals once a week over a period of 12 sessions (3 months). The initial irradiation dose was 100–200 mJ, depending on skin type. If no adverse events occurred, the irradiation dose was increased by 100 mJ at each session. Once the erythema persisted for 24 to 48 hours, the dose was kept constant. If the erythema persisted for more than 48 hours or was accompanied by itching, the dose was reduced by 100 mJ in the next session. If the erythema became painful or was accompanied by vesicles or blisters, further treatment was postponed. ILC‐injections (triamcinolone 5 mg/mL) were administered every 4 weeks in sessions 1, 5 and 9 (3 times); generally, 0.1–0.2 mL per square centimetre of affected skin was injected. Patients returned to the clinic for a follow‐up examination one month after treatment. Excimer laser therapy is inferior to ILC therapy in terms of efficacy. In terms of the endpoint of hair regrowth of more than 50%, 47% of AA patches treated with laser achieved a positive response to therapy compared to 66% of patches treated with ILC (OR 0.45, p = 0.088), but the difference is not statistically significant.^53^

Relapses were not evaluated in some of the studies. One study on the 308 nm excimer laser reported that an average improvement of 47.5% was achieved after 3 months, but only 23.7% improvement after 6 months. The authors attributed this reduction to relapses.^60^

The meta‐analysis by Shen et al. 2023 includes 14 studies (10 RCTs and 4 prospective clinical trials) from 2014 to 2022. The studies originate primarily from China, but also from Egypt, Belgium and Turkey.^54^ In total, data from 890 patients with AA are included, with an average age in the studies between 24 and 43 years. Data from 12 studies are included in the meta‐analysis. No information on the severity of AA is reported.

The systematic review by Behrangi et al. 2022b summarises a total of 10 studies from 1996‐2019 involving a total of 329 children with an average age of 9.3 years.^25^ Of these, three are retrospective studies, one is a prospective study, one is an RCT, one is an open‐label study and one is a cohort study. Three of the included studies could not be assigned to a study design. The included studies examined children with a severe form of AA: AT, AU, multilocal AA, subtotal AA and AA ophiasis, or children with AA with >30‐50% hair loss. The following types of laser treatment were used: low‐level light/laser therapy (LLLT), 308 nm excimer lamp, with a minimum intensity of 50 MJ/cm2. The treatments were carried out twice a week for a maximum of 24 sessions. Two studies in the review, involving a total of 29 children, investigated the effectiveness of the excimer laser. Complete hair regrowth was achieved in 63.6% of children in one of the two studies, while in the second study, 41.5% achieved hair regrowth (without a more precise definition). In both studies, the follow‐up period was six months and there was no recurrence of AA in either study in that time. Adverse events reported included erythema, hyperpigmentation, itching and mild skin peeling in 4/11 children.

### Special notes

Laser therapy is a non‐invasive treatment option for AA. There is evidence of the effectiveness of laser and excimer light therapy for AA, but the expert group discussed this therapeutic option very critically due to the UV radiation exposure of excimer therapy, particularly in children and adolescents. Therefore, they decided in favour of a conditional recommendation, even with the available evidence. In addition, most studies have small case numbers, short follow‐up periods and heterogeneous treatment protocols. Further well‐designed randomised controlled trials with larger patient cohorts and long‐term follow‐up are needed to confirm the results and define the optimal treatment parameters.

Low‐level laser therapy (LLLT) has been shown to be potentially useful in various medical applications, including stimulating hair growth, but there are potential concerns regarding its use. Some studies suggest that LLLT may enhance the proliferation of certain cancer cells, although definitive data are not yet available.^61^, ^62^

Possible risks should therefore be carefully explained during the consultation, especially for patients with a history of malignant skin diseases.

### UVA1 phototherapy and photochemotherapy (PUVA)

**Table:** 

	Recommendation	Recommendation strength
**E54**	UVA1 therapy **should not** be offered to children, adolescents and adults with all forms of AA.	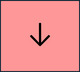
	Strong consensus	**Evidence‐based**
	References: 63, 64, 65, 66	
	LoE: Level 1, Level 2*, Level 2	

**Table:** 

	Recommendation	Recommendation strength
**E55**	PUVA therapy **should not** be offered to children, adolescents and adults with all forms of AA.	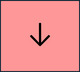
	Majority approval	**Evidence‐based**
	References: 4, 65, 66, 67, 68	
	LoE: Level 2*, Level 2	

Full explanations on efficacy, dosage and special instructions can be found in the long version of the guideline.

### Topical therapy of special localisations

The following overview summarises the recommendations for topical therapy of specific localisations.

**Table:** 

		Recommendation strength
**E56**	In the case of AA of the eyebrows, topical corticosteroids (class II ‐ III, maximum 3 months) or topical calcineurin inhibitors **ought to** be offered in *off‐label‐use* in this area.	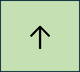
**E57**	For AA of the eyebrows, intralesional corticosteroids (2.5 mg/ml triamcinolone acetonide 0.1ml/cm^2^, strictly intracutaneous) **can** be offered in *off‐label‐use* by physicians experienced in this area.	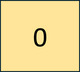
**E58**	For AA of the eyelashes, prostaglandin analogues **can** be offered in *off‐label‐use* in this area.	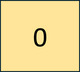
**E59**	For AA barbae, topical corticosteroids (class II ‐ III, maximum 3 months) or topical calcineurin inhibitors **ought to** be offered in *off label use* in this area.	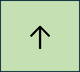
**E60**	For AA barbae, intralesional corticosteroids (2.5 mg/ml triamcinolone acetonide 0.1ml/cm^2^, strictly intracutaneous) **can** be offered in *off‐label‐use* in this area by physicians experienced in their use.	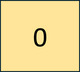
	Strong consensus	**Consensus‐based**

### Systemic therapy

#### Introduction

The various active substances used in systemic therapy for AA are generally not approved for the treatment of AA in Germany but can be used *off‐label*. The two JAK inhibitors baricitinib and ritlecitinib are exceptions. As of 07/2025: For the treatment of severe alopecia areata in adults (>18 years of age), the JAK inhibitor baricitinib has been approved throughout Europe at a dosage of 4 mg/day since 20 June 2022.^69^

Since 15 September 2023, the JAK inhibitor ritlecitinib has also been approved throughout the EU for the treatment of severe alopecia areata in adolescents and adults (>12 years of age) at a dosage of 50 mg/day.^70^ According to Section 34 (1) sentence 7 SBG V, both drugs are eligible for prescription but not reimbursable.

*Off‐label‐use* is indicated in the recommendations. However, it is not mentioned again in the background text of the long version.

According to the Social Security Code (SGB) Book V ‐ Statutory Health Insurance ‐ (Article 1 of the Act of December 20, 1988, Federal Law Gazette I p. 2477), § 34 Excluded Medicines, Remedies, and Aids, all medications that “serve to improve hair growth” are affected by this regulation, which means that statutory health insurance companies do not cover the costs of these medications in the treatment of AA.^3^ To have the costs covered for children, adolescents, and adults, individual applications for reimbursement can be submitted to the health insurance companies. However, due to the above‐mentioned legal situation, the vast majority of individual applications for reimbursement are rejected by health insurance companies.

The guideline group has approved the following general therapy principles for systemic therapy.

### General principles of therapy

**Table:** 

		Recommendation strength
**E6**	The initiation of systemic therapy **ought to** be offered **to adults** with AA in the following situations:	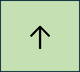
	‐ Patients with moderate AA and above who have not responded to topical treatment options for 6 months	
	‐ For severe to very severe AA lasting longer than six months	
	‐ For rapidly progressive AA within days to weeks	
	‐ Patients with moderate AA and above with high psychological distress / pronounced restriction of quality of life	
	‐ For AA with severe nail involvement	
	Strong consensus	

**Table:** 

		Recommendation strength
**E7**	The initiation of systemic therapy **ought to be** offered **to adolescents** with AA in the following situations:	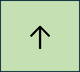
	‐ From moderate AA with high level of suffering / pronounced reduction in quality of life in the absence of a response to topical treatment options over 6 months	
	‐ For severe to very severe AA with a duration of more than 6 months	
	‐ For rapidly progressive AA within days to weeks	
	Strong consensus	

**Table:** 

		Recommendation strength
**E8**	**In children** with AA, systemic therapy **ought to** be used in selected individual cases after special consideration of the benefits and risks.	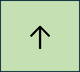
	Consensus	

**Table:** 

		Recommendation strength
**E9**	If there is an insufficient response to topical or systemic therapy over a period of 6 months, the therapeutic approach **should** be re‐evaluated and a switch to an alternative therapy offered.	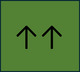
	Strong consensus	

Table 2 shows all systemic treatment options, separated by age group and severity of AA with different levels of recommendation. As only four treatment options were given a “can” or “should” recommendation, only these four treatments are described in more detail below. All other treatments are not recommended based on the analysed literature and the expert assessment; the detailed justification for each agent can be found in the long version of the guideline.

**TABLE 2:** Overview of all recommendations by active substance ‐ Systemic therapy

Active substance	SALT‐Score (Severity)	Children	Adolescents	Adults
**Pulse therapy with Corticosteroids**	Mild <25%	**↓**	**↓**	**↓**
	Moderate 25‐49%	**↓**	**↓**	**0**
	Severe >50‐75%	**0**	**0**	**↑**
	Very severe 76% ‐ 100%	**0**	**0**	**↑**
**Methotrexate (MTX)**	Mild <25%	**↓**	**↓**	**↓**
	Moderate 25‐49%	**↓**	**↓**	**0** ^*^
	Severe >50‐75%	**0**	**0**	**0** ^*^
	Very severe 76% ‐ 100%	**0**	**0**	**0** ^*^
**Cyclosporin A**	Mild <25%	**↓↓**	**↓↓**	**↓↓**
	Moderate 25‐49%	**↓↓**	**↓↓**	**↓↓**
	Severe >50‐75%	**↓↓**	**0** ^**^	**0** ^**^
	Very severe 76% ‐ 100%	**↓↓**	**0** ^**^	**0** ^**^
**Tumour necrosis factor (TNF) α‐inhibitors**	Mild <25%	**↓↓**	**↓↓**	**↓↓**
	Moderate 25‐49%	**↓↓**	**↓↓**	**↓↓**
	Severe >50‐75%	**↓↓**	**↓↓**	**↓↓**
	Very severe 76% ‐ 100%	**↓↓**	**↓↓**	**↓↓**
**Monoclonal antibodies, for example dupilumab**	Mild <25%	No recommendation	No recommendation	No recommendation
	Moderate 25‐49%	No recommendation	No recommendation	No recommendation
	Severe >50‐75%	No recommendation	No recommendation	No recommendation
	Very severe 76% ‐ 100%	No recommendation	No recommendation	No recommendation
**Azathioprine**	Mild <25%	No recommendation	No recommendation	No recommendation
	Moderate 25‐49%	No recommendation	No recommendation	No recommendation
	Severe >50‐75%	No recommendation	No recommendation	No recommendation
	Very severe 76% ‐ 100%	No recommendation	No recommendation	No recommendation
**Januskinase inhibitors** (Baricitinib >18 ys, Ritlecitinib >12 ys, Status 07/2025	Mild <25%	No recommendation	No recommendation	No recommendation
	Moderate 25‐49%	No recommendation	No recommendation	No recommendation
	Severe >50‐75%	**0** ^***^	**0** ^***^	**↑**
	Very severe 76% ‐ 100%	**0** ^***^	**0** ^***^	**↑**
**Glycyrrhizin**	Mild <25%	**↓↓**	**↓↓**	**↓↓**
	Moderate 25‐49%	**↓↓**	**↓↓**	**↓↓**
	Severe >50‐75%	**↓↓**	**↓↓**	**↓↓**
	Very severe 76% ‐ 100%	**↓↓**	**↓↓**	**↓↓**

* = Systemic therapy with methotrexate **should** be offered to adults with a moderate, severe or very severe form of AA as combination therapy with systemic corticosteroids

** = Systemic therapy with cyclosporine A **can** be offered to adolescents and adults with a severe or very severe form of AA with a rapidly progressive course for a short‐term therapeutic intervention.

*** **=** Systemic therapy with JAK inhibitors **can** be offered to children and adolescents with severe and very severe AA with appropriate authorisation for the age group.

Status 07/2025: Since 20 June 2022, the JAK inhibitor baricitinib has been approved throughout Europe at a dosage of 4 mg/day for the treatment of severe alopecia areata in adults (>18 years of age) (1).

Since 15 September 2023, the JAK inhibitor ritlecitinib at a dosage of 50 mg/day has also been approved throughout the EU for the treatment of severe alopecia areata in adolescents and adults (>12 years of age) (2).

According to Section 34 (1) sentence 7 SBG V, both drugs are eligible for prescription but not reimbursable.

### Systemic corticosteroids

**Table:** 

		Recommendation strength
**E61**	If systemic corticosteroid therapy is offered for the treatment of AA, it **ought to** be administered in pulsed doses and not, as a rule, continuously in *off‐label‐use*.	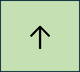
	Strong consensus	**Evidence‐based**
	References: ^63^	
	LoE: Level 1	

**Table:** 

	Recommendation	Recommendation strength
**E62**	Systemic corticosteroids **should not** be offered to children and adolescents with a mild or moderate form of AA and adults with a mild form of AA.	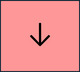
	Strong consensus	**Evidence‐based**
	References: 63, 71	
	LoE: Level 1, Level 2	

**Table:** 

	Recommendation	Recommendation strength
**E63**	Systemic corticosteroids **can** be offered to children and adolescents with a severe or very severe form of AA in *off‐label‐use*.	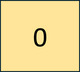
	Strong consensus	**Evidence‐based**
	References: 4, 71	
	LoE: Level 2*, Level 2	

**Table:** 

	Recommendation	Recommendation strength
**E64**	Systemic corticosteroids **can** be offered to adults with a moderate form of AA in *off‐label‐use*.	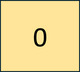
	Strong consensus	**Evidence‐based**
	References: ^63^	
	LoE: Level 1	

**Table:** 

	Recommendation	Recommendation strength
**E65**	Systemic corticosteroids **ought to** be offered to adults with a severe or very severe form of AA in *off‐label‐use*.	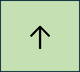
	Strong consensus	**Evidence‐based**
	References: 29, 63	
	LoE: Level 1	

The full explanations on efficacy, dosage and special instructions can be found in the long version of the guideline.

### Methotrexate

**Table:** 

	Recommendation	Recommendation strength
**E66**	Systemic therapy with methotrexate **should not** be offered to children and adolescents with a mild or moderate form of AA and adults with a mild form of AA.	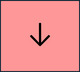
	Strong consensus	**Evidence‐based**
	References: 72, 73	
	LoE: Level 2*	

**Table:** 

	Recommendation	Recommendation strength
**E67**	Systemic therapy with methotrexate **can** be offered to children and adolescents with a severe or very severe form of AA in *off‐label‐use*.	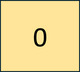
	Strong consensus	**Evidence‐based**
	References: 72, 73	
	LoE: Level 2*	

**Table:** 

	Recommendation	Recommendation strength
**E68**	Systemic therapy with methotrexate **can** be offered to adults with a moderate, severe or very severe form of AA as monotherapy in *off‐label‐use*.	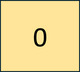
	Strong consensus	**Evidence‐based**
	References: 72, 73	
	LoE: Level 2*	

**Table:** 

	Recommendation	Recommendation strength
**E69**	Systemic therapy with methotrexate **ought to** be offered to adults with a moderate, severe or very severe form of AA as a combination therapy with systemic corticosteroids in *off‐label‐use*.	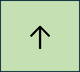
	Strong consensus	**Evidence‐based**
	References: 72, 73, 74, 75	
	LoE: Level 2*, Level 2	

The full explanations on efficacy, dosage and special instructions can be found in the long version of the guideline.

### Excerpt from mechanism of action and efficacy

There is international consensus on the following recommendation for treatment with MTX: In adults, the target dose of MTX should be 15 to 25 mg per week. MTX is suitable for the treatment of severe and very severe AA in adolescents. If MTX therapy is started in patients under 18 years of age with severe AA, the target dose should be 0.4 mg/kg/week.^76^ Rudnicka et al. 2024 agreed that steroid‐sparing agents such as MTX should only be used to reduce the risk of side effects of systemic corticosteroid therapy when planned as prolonged use.^77^

In the meta‐analysis by Phan et al. 2019, a complete response to MTX therapy of 35.8% (95% CI 25.0%‐48.3%) was calculated across all studies. The included studies only included patients with a severe form of AA who had either at least a SALT score of >50% or an AT or AU. In adults only (n = 315), the proportion of patients with a complete response to therapy was 44.7% (95% CI 32.9%‐57.1%), in children (n = 58) this proportion was 11.6% (95% CI 5.1%‐24.5%).^72^ When comparing the combination of MTX plus systemic corticosteroids versus stand‐alone MTX therapy, the results of the meta‐analysis (OR 2.73, 95% CI 1.19‐6.27),^72^ but also of a small randomised controlled three‐arm study (methotrexate vs. betamethasone pulse therapy vs. combination) showed superiority of the combination therapy (SALT score reduction of 43% three months after six months of therapy) compared to the monotherapies (SALT score reduction of 26% with betamethasone and 23% with MTX).^74^

The average time from diffuse regrowth to complete hair regrowth was between 3.3 months (95% CI 2.3 ‐ 4.0) and 9.9 months (95% CI 6.0‐13.8) in the studies in the meta‐analysis by Phan et al. 2019. Across all studies with children and adults, the recurrence rate (mostly after dose reduction or discontinuation of therapy) was 47.7% (95% CI 35.2%‐60.5%) for all patients, 52.0% (95% CI 37.5%‐66.2%) for adults and 31.7% (95% CI 16.3%‐52.6%) for children.^72^

### Combination with other therapies

MTX therapy at the above dosage can be combined in adults and children with the simultaneous systemic administration of steroids,^4^, ^72^ (high doses at the start of MTX treatment; gradually discontinued within one to two weeks (for more details, see section “Systemic corticosteroids”) or with topical anti‐inflammatory therapy.

Interactions with MTX with increased bone marrow and liver toxicity are possible when taken concomitantly with sulphonamides, barbiturates, tetracyclines, retinoids, salicylates and ethanol.

### Special notes

MTX is teratogenic, so an effective contraceptive method is essential for adolescent females and women of childbearing age during treatment and for up to six months after completion of therapy. The same applies to men who are treated with MTX. Breastfeeding during treatment with MTX is not recommended.

Vaccination with live vaccines should not be carried out during therapy. Further vaccinations should only be given after consultation with the treating doctor.

Absolute contraindications for treatment with MTX are severe renal dysfunction, liver damage, diseases of the haematopoietic system, alcohol abuse, immunodeficiency, severe infections, gastrointestinal ulcers and malignant lymphomas.

### Cyclosporin A (calcineurin inhibitor)

**Table:** 

	Recommendation	Recommendation strength
**E70**	Systemic therapy with cyclosporine A **shall not** be offered to children with all forms of AA and adolescents and adults with a mild or moderate form of AA.	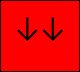
	Strong consensus	**Evidence‐based**
	References: 4, 28, 78	
	LoE: Level 2*, Level 2	

**Table:** 

	Recommendation	Recommendation strength
**E71**	Systemic therapy with cyclosporine A **can** be offered to adolescents and adults with a severe or very severe form of AA with a rapidly progressive course for a short‐term therapeutic intervention in *off‐label‐use*.	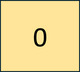
	Strong consensus	**Evidence‐based**
	References: 4, 28, 78	
	LoE: Level 2*, Level 2	

The full explanations on efficacy, dosage and special instructions can be found in the long version of the guideline.

### Monoclonal antibodies

#### Tumour necrosis F alpha inhibitors (TNF‐alpha inhibitors)

**Table:** 

	Recommendation	Recommendation strength
**E72**	Systemic therapy with tumour necrosis factor (TNF) α inhibitors **shall not** be offered to children, adolescents and adults with all forms of AA.	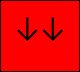
	Strong consensus	**Evidence‐based**
	References: ^79^	
	LoE: Level 2*	

Full explanations on efficacy, dosage and special instructions can be found in the long version of the guideline.

### Various monoclonal AK, modified cytokine and recombinant protein

**Table:** 

	Statement	
**S4**	Based on the evidence identified, the Guideline Group cannot make a recommendation for or against the use of monoclonal antibodies (except TNF‐alpha inhibitors, see E72) that have been investigated in trials for the treatment of AA in children, adolescents and adults.	
	Strong consensus	**Evidence‐based**
	References: 49, 80, 81	
	LoE: Level 1, Level 2*, Level 2	

Full explanations on efficacy, dosage and special instructions can be found in the long version of the guideline.

### Azathioprine

**Table:** 

	Statement	
**S5**	Based on the evidence identified, the Guideline Group cannot recommend the use of azathioprine for the treatment of AA in children, adolescents and adults.	
	Consensus	**Evidence‐based**
	References: 4, 29, 82, 83	
	LoE: Level 1, Level 2*, Level 2	

Full explanations on efficacy, dosage and special instructions can be found in the long version of the guideline.

### Januskinase inhibitors (JAK inhibitors)

**Table:** 

	Recommendation	Recommendation strength
**E73**	Systemic therapy with JAK inhibitors **can** be offered to children and adolescents with severe and very severe AA with the appropriate authorisation for the age group.	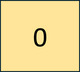
	Strong consensus	**Evidence‐based**
	References: 44, 84, 85, 86	
	LoE: Level 1, Level 2*	

**Table:** 

	Recommendation	Recommendation strength
**E74**	Systemic therapy with JAK inhibitors **ought to** be offered to adults with severe and very severe AA.	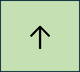
	Strong consensus	**Evidence‐based**
	References: 29, 43, 85, 86, 87	
	LoE: Level 1, Level 2*	

For the treatment of severe AA in adults (>18 years of age), JAK inhibitor baricitinib has been approved throughout Europe at a dosage of 4 mg/day since 20 June 2022.^69^

Since 15 September 2023, the JAK inhibitor ritlecitinib in a dosage of 50 mg/day has also been approved throughout the EU for the treatment of severe AA in adolescents and adults (>12 years of age).^70^

According to Section 34 (1) sentence 7 SBG V, both medications can be prescribed but are not reimbursable.

The full explanations on efficacy, dosage and special instructions can be found in the long version of the guideline.

### Excerpt from mechanisms of action and efficacy

Janus kinase inhibitors such as baricitinib, ritlecitinib, tofacitinib, deuruxolitinib, ruxolitinib and brepocitinib are small molecules, which can inhibit intracellular cytokine transduction by blocking the JAK‐STAT signalling pathway (JAK‐signal transducers and activator of transcription (STAT)) and thus have an immunomodulatory, antiproliferative and anti‐inflammatory effect.^88^ Some JAK inhibitors inhibit selectively (upadacitinib and abrocitinib (JAK 1), brepocitinib (JAK 1/TYK 2), ritlecitinib (JAK 3 and TEC) and others less selectively (tofacitinib (JAK 1,3>2), deuruxolitinib, ruxolitinib and baricitinib (JAK 1, 2), which is based on their isotype specificity (depending on ATP binding region). Based on their binding methods and interactions, JAK inhibitors can also be subclassified into reversible (competitive) and irreversible (covalent) inhibitors (such as ritlecitinib).^88^

In the Cochrane review by Mateos‐Haro et al. 2023, a risk ratio RR 7.54 (95% CI 3.90 ‐ 14.58) was calculated for the endpoint >75% hair regrowth as a short‐term endpoint (between twelve‐ and 26‐weeks follow‐up), which showed clear superiority compared to placebo therapy. This clear superiority (RR 8.49 (95% CI 4.70 ‐ 15.34),^29^ was also shown for the endpoint of >75% hair regrowth as a long‐term endpoint (>26 weeks follow‐up).

The pooled results of the meta‐analysis by Mao et al. 2023 (from 5 RCTs with a total of 1455 patients from clinical trials from 2016‐2022; AA types not always reported) with different JAK inhibitors (baricitinib, ritlecitinib, tofacitinib, ruxolitinib, ATI‐501) show that treatment with JAK inhibitors achieved a fivefold increase in the probability of achieving SALT score 50 (OR 5.08 (95% CI, 3.49‐ 7.38) compared to placebo treatment.^85^ The probability of achieving the SALT 90 score was even higher (OR 7.40 (95% CI, 4.34‐12.67).^85^

Information on the efficacy and safety of individual JAK inhibitors (baricitinib, deuruxolitinib, brepocitinib, ritlecitinib, tofacitinib) in AA patients is provided by the systematic review by Sedeh et al. 2023, which includes 37 studies (with meta‐analysis from 5 RCTs; average age of patients from 4.3 to 66 years) from 2016‐2022,^86^ and the meta‐analysis by Wei D et al. 2023 with four RCTs from 2019 to 2022 with a total of 1689 patients.^87^

Oral baricitinib was shown to be more effective than the other JAK inhibitors in patients with any form of AA. Baricitinib 4 mg once daily is superior to other treatment options brepocitinib and ritlecitinib in three studies (n = 357) with endpoint SALT_(50)_ (baricitinib: risk difference (RD) 63%, 95% CI 44‐82 vs. brepocitinib: RD 47, 95% CI 32‐62; and ritlecitinib: RD 33%, 95% CI 19‐47) and endpoint SALT_(75)_ (baricitinib: RD 45%, 95% CI 25‐65 compared to brepocitinib: RD 38%, 95% CI 24‐53 and ritlecitinib: RD 25%, 95% CI 12‐38).^86^

For patients with severe AA (SALT score > 50), a superiority for oral deuruxolitinib 12 mg 2 x tgl. in two studies where the achievement of the SALT_50_score (deuruxolitinib 12 mg: RD 48%, 95% CI 30‐66 compared to baricitinib 4 mg: RD 42%, 95% CI 35‐49) and the SALT_(75)_ (deuruxolitinib 12 mg: RD 34%, 95% CI 16‐51 compared to baricitinib 4 mg: RD 31%, 95% CI 25‐37) was investigated.^86^

The systemic review by Yan et al. 2022 (with 14 studies from 2016‐2021 with a total of n = 1845 patients with AA, AU, or AT aged 27.8 to 47.6 years) shows clear superiority of JAK inhibitors not only over other treatment options (good response in RCTs defined as SALT_50_values: RR: 6.86; (95% CI: 2.91‐16.16) and in observational studies 63% (95% CI: 44%‐80%)), but also over JAK inhibitors administered topically (ruxolitinib or tofacitinib) or sublingually (tofacitinib). There was no statistically significant difference in the subgroup analysis of topically administered JAK inhibitors compared to the control group (RR 1.00, 95% CI: 0.31‐3.18 in the RCTs). The observational studies showed that 28% of patients (95% CI: 1%‐72%) who used a topically administered JAK inhibitor and 11% (95% CI: 1%‐29%) who used a sublingually administered JAK inhibitor responded well to the therapy. The proportion of patients in whom AA recurred (recurrence rate) was 54% (95% CI: 39%‐69%). The main cause of disease recurrence was discontinuation of JAK inhibitors.^43^

JAK inhibitors have also been shown to be effective and safe in children and adolescents with AA. In the meta‐analysis by Chen et al. 2022 with ten studies from 2017‐2022 with a total of 69 patients aged 3‐18 years with AA, AU or AT and a disease duration of 1‐15 years, a general response (“any positive response”) to JAK inhibitor therapy (tofacitinib oral or topical and ruxolitinib oral) of 81.9% (95%CI: 69.9‐91.9%) was calculated.^44^ Oral tofacitinib showed an average overall hair regrowth rate of 49% (95% CI 29%‐69%) in 59 patients aged 3 to 18 years with AA, AT and AU.^44^ The meta‐analysis by Behrangi et al. 2022a (seven studies on tofacitinib from 2017‐2021) reported that 19% (95% CI 6%‐36%) had little to no regrowth and 12% (95% CI 1%‐29%) had an increase in AA disease during treatment.^84^

### Excerpt from safety

The most common adverse effects with JAK inhibitors (baricitinib, ritlecitinib, ATI‐501, tofacitinib, ruxolitinib) in AA patients were infections, abnormal laboratory values, neurological symptoms, gastrointestinal reactions and skin symptoms.^85^

Sedeh et al. 2023 reported the side effects of individual JAK inhibitors in detail. Respiratory tract infections, nasopharyngitis, urinary tract infections and herpes zoster infections were reported most frequently with tofacitinib, baricitinib and ruxolitinib. Upper respiratory tract infections and nasopharyngitis are also common with deuruxolitinib, ritlecitinib and brepocitinib. Headaches predominate with deuruxolitinib, baricitinib, ritlecitinib and Brepocitinib.^86^

Cutaneous events such as acne, acneiform eruptions and folliculitis have occurred with most JAK inhibitor therapies (tofacitinib, deuruxolitinib, baricitinib, delgocitinib, ruxolitinib, ritlecitinib) (acne 3 times more frequent with baricitinib compared to control group (RR 3.48; 95% CI 1.55‐7.82).^43^ Nausea and/or diarrhoea have been reported with deuruxolitinib, baricitinib, ritlecitinib and brepocitinib. An increase in creatine phosphokinase or creatine kinase in the blood has been reported with baricitinib and deuruxolitinib, herpes simplex infections as an undesirable side effect particularly with baricitinib. Weight gain, fatigue are primarily reported with ruxolitinib and conjunctivitis, increase in alanine transaminase (ALT), aspartate aminotransferase (AST), cholesterol and triglycerides (in children also eosinophilia (n = 5/49, 10.2%),^44^ with tofacitinib alone. Blood count changes in the sense of leucopenia occur during treatment with tofacitinib and ruxolitinib.^86^

The systematic review by Papierzewska et al. 2023 reports that hyperseborrhoea (4.1%) occurred sporadically with tofacitinib, hypercholesterolaemia and cellulitis with baricitinib and rhabdomyolysis with Brepocitinib.^89^ In two phase 3 studies with baricitinib vs. placebo, serious adverse events occurred in n = 22 patients (2.4% vs. 1.6%, OR 1.5) in the sense of acute myocardial infarction (0.1% vs. 0%, OR 1.23), ventricular tachycardia (0.1% vs. 0%, OR 1.23), hypertension (0.1% vs. 0%, OR 1.23), acute cholecystitis (0.2% vs. 0.3%, OR 0.8), pyelonephritis (0.2% vs. 0%, OR 2.1), bone fractures (0.7% vs. 0.3%, OR 2.5), B‐cell lymphoma (0.1% vs. 0%, OR 1.23), Guillain‐Barré syndrome (0.1% vs. 0%, OR 1.23), abortion (0.1% vs. 0%, OR 1.23). No deaths were reported.^89^

During the period of the studies from 2016‐2022 in the meta‐analysis by Mao et al. 2023, no cases of malignancy or tuberculosis occurred under JAK inhibitor therapy,^85^ however, the treatment period in the included clinical studies was only between 24 and 36 weeks and in the observational studies between 4 and 42 months. Under baricitinib, one case (n = 1/22) with B‐cell lymphoma (0.1% vs. 0%, OR 1.23),^89^ and in the BRAVE‐AA2‐trial one squamous cell carcinoma (0.5%, IR = 0.5) after 16 months with baricitinib 2 mg and one ductal carcinoma in situ (0.4%, IR = 0.3) after ten months with baricitinib 4 mg occured.^69^

Two malignancies were reported with ritlecitinib in the phase 2b‐3 study: Breast cancer in the 50 mg group described in association with treatment according to the investigator and breast cancer in the 200 mg + 50 mg group that was not seen in association with treatment.^70^

Oral baricitinib, tofacitinib and ruxolitinib treatment had significantly lower rates of treatment‐related adverse events compared to conventional corticosteroid treatment (MD 0.08, 95% CI 0.02‐ 0.42; MD 0.14, 95% CI 0.04‐ 055; MD 0.35, 95% CI 0.14‐ 0.88).^87^

Adolescents who have no history of chickenpox should be vaccinated against chickenpox, the experts explicitly stated.^90^ A herpes zoster (HZ) vaccination is also recommended for adults before initiating treatment with JAKi, even if this vaccination is only reimbursed by health insurance companies from the age of 60; only people at increased risk can be reimbursed from the age of 50. The HZ vaccination is authorised from the age of 18.

### Monitoring

Before treatment with JAK inhibitors, screening for chronic infections by laboratory (e.g. hepatitis B‐/C, HIV, tuberculosis) and radiological (chest X‐ray) methods is required. The vaccination status must be checked. Before and during therapy, laboratory checks of the differential blood count, liver and kidney values and blood lipid values (cholesterol, LDL, HDL, triglycerides) should be carried out at regular intervals in accordance with the information for healthcare professionals.

### Further steroid‐sparing therapies

**Table:** 

	Recommendation	Recommendation strength
**E75**	Systemic therapy with glycyrrhizin **shall not** be offered to children, adolescents and adults with all forms of AA.	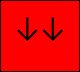
	Strong consensus	**Evidence‐based**
	References: 91, 92, 93	
	LoE: Level 2	

Full explanations on efficacy, dosage and special instructions can be found in the long version of the guideline.

### Active substances that have proven ineffective in the treatment of alopecia areata

#### Dapsone

In the treatment of AA, there have been numerous attempts to identify active substances that can be used to treat AA successfully. As early as 1981 and 1995, for example, two publications reported that the active substance dapsone was ineffective.^94^, ^95^

#### Etacercept/Alefacept

Strober et al. 2005 describe that after continuous treatment with 50 mg etanercept, administered subcutaneously twice a week over a period of 8 to 24 weeks, none of the treated subjects showed significant hair regrowth. Based on these results, the authors conclude that etanercept appears to be ineffective in the treatment of subjects with therapy‐resistant, moderate to severe alopecia areata, alopecia totalis or alopecia universalis.^96^

Strober et al. 2009, in their multicentre, double‐blind, randomised, placebo‐controlled clinical trial, evaluated the efficacy of alefacept in the treatment of severe alopecia areata (AA). They included 45 individuals with chronic and severe AA affecting 50% to 95% of the scalp hair and unresponsive to previous therapies in their study. Alefacept, a biological T‐cell inhibitor approved by the US Food and Drug Administration (FDA) for the treatment of moderate to severe plaque psoriasis, was to be tested for its efficacy in AA. The outcome measure was an improved SALT score over 24 weeks. Participants who received alefacept for 12 weeks showed no statistically significant improvement in AA compared to the placebo group (p = 0.70). The authors concluded that alefacept is ineffective for the treatment of severe AA.^97^

The current British guideline on AA by Harries et al. 2025 makes statements consistent with the recommendations of this guideline on other active substances that have shown negative results once or repeatedly in the treatment of AA.^98^

“There is **insufficient evidence** to recommend the following **systemic interventions** for people with AA”: Inosiplex (isoprinosine or inosine pranobex), imipramine, apremilast, sulfasalazine, mesalazine, hydroxychloroquine, dimethyl fumarate.^98^

According to the British guideline, there is also insufficient evidence for non‐steroidal injectable therapies:^98^ platelet‐rich plasma (PRP), microneedling, carboxytherapy, cryotherapy, intralesional pentoxifylline injection, intralesional methotrexate injection, intralesional vitamin D injection, mesenchymal stem cells, intralesional interferon alpha injection, intradermal minoxidil injection.

In addition, the British guideline states that there is insufficient evidence to recommend the following alternative interventions for people with AA: aromatherapy, allium/onion ointment, ginseng, peony, glycyrrhizin, combined complementary therapies (herbs, nettle, dandelion), toxic primrose (*Primula obconica*), herbal sensitizers, candida antigen, squill lotion, and hypnosis.^98^

### Supportive administration of food supplements

**Table:** 

	Statement	
**S6**	Based on the evidence identified, the Guideline Group **cannot** recommend the use of dietary supplements for the treatment of AA in children, adolescents and adults.	
	Strong consensus	**Evidence‐based**
	References: 99, 100	
	LoE: Level 2*	

The full explanations on efficacy, dosage and special instructions can be found in the long version of the guideline.

### Psychological support

**Table:** 

	Recommendation	Recommendation strength
**E76**	All patients with all forms of AA, regardless of age, **should** be informed about the possibilities of psychological support.	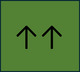
	Strong consensus	**Consensus‐based**

**Table:** 

	Recommendation	Recommendation strength
**E77**	All patients with all forms of AA, regardless of age, **should** be informed about the services offered by self‐help groups.	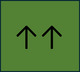
	Strong consensus	**Consensus‐based**

**Table:** 

	Recommendation	Recommendation strength
**E78**	If there is a positive history of depression or social anxiety, a psychotherapeutic diagnosis and/or supportive psychotherapy **ought to** be recommended.	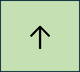
	Strong consensus	**Consensus‐based**

The full explanations on can be found in the long version of the guideline.

### Quality of life during therapy

Studies on the quality of life of AA patients consistently show that their quality of life is impaired but improves with treatment. For example, quality of life results of successfully treated patients with AA in Australia are like those of the Australian population. In adults, there is a clear correlation with the success of treatment: the more hair regrows, the more the quality of life improves.^75^, ^101^, ^102^, ^103^ The quality of life improved most significantly for patients who achieved a SALT score <20 during treatment.^104^

Therapies that do not affect hair regrowth, such as hypnotherapy or mindfulness‐based stress reduction, also improve quality of life.^105^

For children, AA disease also means a reduction in their quality of life, which is even significantly higher compared to healthy peers. The systematic review by Prendke et al. 2023 reports a low to moderate impairment of the quality of life of children and adolescents due to AA. The measured mean values of the Childrens Dermatology Life Quality Index (cDLQI) were 2.2 (SD 0.96) or 4.4 (SD 4.9) with a range of 0‐22 or 6.3 (SD 5.9) in the included studies. There was no clear correlation between treatment success and quality of life in children, but the quality of life of adolescents with AA often deteriorates with the onset of puberty.^106^

### Cosmetic services

**Table:** 

	Recommendation	Recommendation strength
**E79**	All patients with all forms of AA, regardless of age, **should** be informed about the possibilities of hair replacement and cosmetic options.	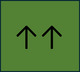
	Strong consensus	**Consensus‐based**

The expert panel is in favour of providing general information on cosmetic services and hair replacement for all patients to highlight the full range of management and disease management options from the outset. This should help patients to cope with the disease and allay or reduce fears and concerns about possible developments during AA at an early stage.

Further details, specifically information on which options dermatologists can draw attention to, can be found in tabular form in the long version.

## CONFLICTS OF INTEREST STATEMENT

A complete list of declared conflicts of interest can be found in the guideline report on *the AWMF website* https://register.awmf.org/de/leitlinien/detail/013‐104.
